# How membranes shape up for lipid transfer

**DOI:** 10.7554/eLife.111373

**Published:** 2026-05-07

**Authors:** Takashi Hirashima, Toshiya Endo

**Affiliations:** 1 Faculty of Life Sciences and the Protein Dynamics Institute, Kyoto Sangyo University Kyoto Japan

**Keywords:** mitochondria, lipid transport, phosphatidic acid, cardiolipin, None

## Abstract

The extraction of a phospholipid called phosphatidic acid from the mitochondrial outer membrane is regulated by the curvature of this membrane.

**Related research article** Sadeqi F, Dong D, Stroh K, Vache M, Metz J, Riedel D, Janshoff A, Risselada HJ, Kolenda C, Meinecke M. 2026. Membrane curvature regulates Ups1 dependent phosphatidic acid transfer across lipid bilayers. *eLife* **14**:RP106979. doi: 10.7554/eLife.106979.

Biological membranes are masterpieces of cellular architecture. Composed of lipid bilayers and proteins, they form protective yet permeable barriers around cells. These membranes can also display a wide range of curvatures, both positive (convex) and negative (concave), with the amount of curvature depending on external forces, interactions between the lipids and the proteins, and differences in the composition of the two layers of the bilayer (Jarsch et al., 2016; Johnson et al., 2024). This raises a fundamental question: what is the functional significance of membrane curvature?

The organelles inside cells – such as the nucleus and mitochondria – are also enclosed by membranes made of a double layer of specific phospholipids tailored to their function. For example, mitochondria have both an outer membrane and an inner membrane containing cardiolipin. This phospholipid has an essential role in the activity of mitochondria, and defects in cardiolipin have been linked to various heart diseases, metabolic disorders, neurodegenerative diseases and cancer. Cardiolipin is synthesized from another phospholipid – phosphatidic acid – in the inner membrane (Tamura et al., 2020). However, since phosphatidic acid is produced outside mitochondria, it must be transported through the outer membrane to reach the inner membrane.

In yeast, the transfer of phosphatidic acid from the outer membrane to the inner membrane is facilitated by two proteins: Ups1, a lipid-binding protein, and Mdm35, which stabilizes Ups1 and protects it from degradation (Connerth et al., 2012). Previous research suggests that the Ups1–Mdm35 complex transports phosphatidic acid between the outer and inner mitochondrial membranes. Ups1 first binds to the outer membrane and extracts phosphatidic acid (Tamura et al., 2020). It then forms a transient complex with Mdm35 and travels through the aqueous intermembrane space. Upon reaching the inner membrane, Ups1 dissociates from Mdm35, binds to the membrane, and releases the phosphatidic acid (Tamura et al., 2020). Now, in eLife, Michael Meinecke of Heidelberg University and colleagues – including Fereshteh Sadeqi and Dexin Dong as joint first authors – report that curvature-dependent mechanisms are important in this transfer process (Sadeqi et al., 2026).

Molecular dynamics simulations performed by Sadeqi et al. indicated that a positive (convex) membrane curvature stabilizes Ups1 binding. This prediction was experimentally validated by analyzing the interactions of Ups1 with unilamellar liposomes (artificial spherical structures in which a lipid bilayer surrounds an aqueous core) of varying sizes. The researchers also showed that Ups1 requires negatively charged lipids for binding, regardless of curvature. Together, these findings indicate that membrane curvature cooperates with electrostatic interactions to promote Ups1 association with membranes.

Sadeqi et al. then tested how membrane curvature affects lipid transfer rates by the Ups1–Mdm35 complex. An elegant experimental design that varied the curvature of the donor and acceptor liposomes revealed that high positive curvature in the membrane of the donor accelerated the transfer of phosphatidic acid, whereas the curvature of the acceptor had little effect. These results suggest that positive curvature in the outer membranes facilitates the removal of phosphatidic acid by Ups1.

To understand why, Sadeqi et al. used molecular dynamics simulations to calculate the free energy of molecules of phosphatidic acid within membranes and found that molecules in regions of positive curvature had higher energies than those in flat regions. This means that it requires less energy for Ups1 to extract phosphatidic acid from regions of membranes with positive curvature (Figure 1).

**Figure 1.**
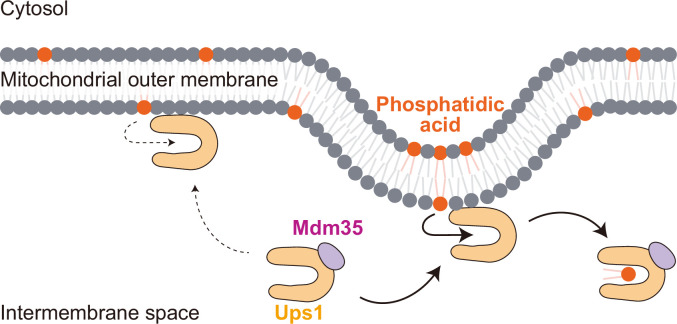
Role of membrane curvature in lipid transfer in mitochondria. Two proteins – Ups1 (light orange horseshoe shape) and Mdm35 (purple) – form a soluble protein complex to transfer a phospholipid called phosphatidic acid (PA; dark orange solid circles) from the outer membrane (top) to the inner membrane (not shown). This process starts with Ups1 binding to outer membrane on its own. Sadeqi et al. show that Ups1 has low affinity for regions of the outer membrane that are flat (left), because PA is in a low-energy state in these regions and is, therefore, difficult to extract. Instead, Ups1 preferentially binds to regions of the outer membrane that have positive curvature (right), because PA is in a high-energy state in these regions, which lowers the energy barrier for extraction, thus enabling efficient lipid transfer. After Ups1 has extracted the PA, the Ups1–Mdm35 complex transfers it to the inner membrane.

So where does this positive curvature in the outer membrane come from Venkatraman et al., 2024? Sadeqi et al. – who are based at Heidelberg University, the Technical University of Dortmund, the University of Göttingen and the Max Planck Institute for Multidisciplinary Science – show that Ups1 binding itself can lead a modest amount of positive curvature. Moreover, certain protein complexes in the outer membrane can also deform it to create positive curvature (Busch et al., 2023). However, it remains unclear how Ups1 can sense membrane curvature, although it might involve recognizing defects in the lipid bilayer. Although the structure of the soluble Ups1–Mdm35 complex is known, high-resolution structures of membrane-associated Ups1 are still lacking (Watanabe et al., 2015), and determining this structure will be key to understanding the molecular basis of both membrane binding and curvature sensing.

The idea that the extraction of phospholipids depends on membrane curvature might also apply to other systems. Because different lipids prefer different curvatures (phosphatidylethanolamine, for example, favors negatively curved membranes), future studies are needed to explore how lipid transfer proteins have evolved their membrane-binding properties to match specific substrates.
